# The Book World of Medicine and Science

**Published:** 1893-05-20

**Authors:** 


### THE BOOK WORLD OF MEDICINE AND SCIENCE.

### BOOK REVIEWS.

DRUNKENNESS. By GEORGE R. WILSON, M.B., C.M., Assistant Physician, the Royal Asylum, Morningside, Edinburgh. (London : Swan Sonnenschein and Co. New York : Charles Scribner's Exors.)

Dr. Wilson's little book on "Drunkenness" is so moderate, thoughtful, and comprehensive that it ought to be in the hands of every charitable worker, and. we may add, of every parent as well. It does not profess to be an original work; it gives no new theories as to the causes and cures of drunkenness, though naturally the author takes the scientific view of alcoholism being a neurotic disease rather than a vice, however truly it is the parent of many vices. But no one who reads it can fail to get a juster and more thoughtful view of the great canker of our national life. Dr. Wilson rates the influence of drunkenness in parents in producing idiocy and insanity in the offspring more highly than does Dr. Potter (in the work on "The Feeble-Minded," which we recently received from the same publishers). He quotes from Dr. Strahan's "Marriage and Disease" the estimates of Dr. Howe, that "50 per cent. of all the idiots in the State of Massachusetts examined by him were the children of intemperate parents"; and of Dr. Fletcher Beach, that to this cause we may assign 25 per cent. of the idiocy received into Darenth Asylum. Dr. Potter "could not make out" more than about 16 per cent. as due to this cause.

At the same time, Dr. Wilson largely accepts Dr. Weissman's theory that parents do not transmit constitutional tendencies to vicious indulgence to their children. What they do hand down is a nervous susceptibility which may ultimately develop into such tendencies. This is not very hopeful, yet is not wholly matter for despair. For it means that the drunkard's children are not inevitably predoomed to their parent's sin, but may be redeemed by being carefully brought up under favourable conditions. The misfortune is that these conditions are not easily to be met with, especially in drunkards' homes. Dr. Wilson lays great emphasis on the sensitiveness of youth to stimulants, especially at the period of adolescence, and advises that no one should touch stimulants under the age of twenty-five. From the wide experience of Dr. Clorston, superintendent of the Royal Asylum at Morningride, he quotes a curious case of a child drunkard. This boy came of a drunken family, and had been of an impulsive and irritable nature from his infancy. When he was about eleven years of age he was given some whisky. The stimulant found a palate ready for it, and at twelve he was a confirmed drunkard. "Ever since that first taste the craving had been present. He stuck at nothing to gratify it; lying and stealing he would practice at any time and get a little of the coveted stimulant. He invented wonderful stories of illness at home, for which whisky was needed at once, messages from his mother to the family tradesmen, &c." Cases like this are fortunately rare, though perhaps less so than is generally supposed. Even where there is no manifest hereditary tendency, a restless, nervous, precocious child should be regarded as an object of extreme care and anxiety. As Dr. Wilson says: "There is a historic order in development which biology inculcates, and which can never be safely ignored, least of all in the case of

THE BOOK WORLD OF MEDICINE AND SCIENCE.—(*continued*).

nervous children. Genius that does not rest on a firm motor and trophic basis is an anomally."

The book is illustrated by many curious anecdotes, showing the different effects of drunkenness on different natures. One young woman, unmarried, not acting on any vague remembrance of love and loss, always stole a child when she was tipsy. In the graphic language of a policeman: "A single instance would not warrant one in concluding that whisky would have this effect upon such a creature, but it most always does upon this woman. When she gets on a regular drunk she always 'cabbages' a young 'un, no matter what sort of one, and caresses it till she sobers up, when she drops it like a hot potato." That curious automatism of memory by which we totally forget an event until circumstances place us in an exactly similar position is paralleled by the case of an Irish porter who delivered a parcel at the wrong house when he was drunk. When he became sober he had totally forgotten where he had left it, but the next time he got drunk he remembered, and went to reclaim it. This is an instance of alcoholic trance, which is paralleled by other morbid conditions. On the moral side a curious case of the influence of will-power on alcoholism is given. A man who was a drunkard at thirty, regarded as hopeless by his friends, suddenly resolved that he would not touch drink until he was seventy. He kept his word, but forty years of abstinence did not destroy the craving. At seventy he began to indulge again, and two years after died of delirium tremens.

In the way of prevention Dr. Wilson believes in the removal of temptation; in prohibition when honestly carried out, and in the establishment of homes where the drunkard might be secluded without the need of obtaining his own consent. He might be reclaimed there, at least he would be prevented from transmitting his vicious tendencies to a new generation. Of course this implies expense. In the case of the well-to-do this need be no hindrance, but with the innumerable drunkards among the poor, would the State supply the need? Why not? We might safely assume a decrease in the need for accommodation in asylums and gaols if the freedom of drunkards were restrained as soon as their disease proclaimed itself; and the general saving of various kinds would be considerable. As Dr. Wilson summarises: "The saving of money, in diminishing the work of the police-court, would be enormous; but the most considerable benefit would be in the general effect of the measure on the conditions of the labouring class and of tradesmen. Bad debts would be much diminished; labour would be improved by the withdrawal of the competition and irregular work of a low class of workmen; money paid as wages would go to men less likely to squander it; charity would be relieved of a class upon whom it now wastes at least half its resources." The problem of drunkenness is one that is pressing for solution. We commend this little book as a very sound and sensible contribution to its consideration.

### THE MAY REVIEWS.

DR. HAMMOND records an interesting series of operations on the brain in the NORTH AMERICAN REVIEW. He writes—"Quite recently operations have been performed upon the

THE BOOK WORLD OF MEDICINE AND SCIENCE—(*continued*).

skull in cases of idiocy, innate or acquired, with the view of removing a supposed disproportion between the size of the brain and the skull, and thus allowing the organ space in which to grow. A French surgeon proposed the removal of strips of the cranium in cases of idiocy in which the skull was unduly small, and in which, as he supposed, there was no room for the brain to expand. Several of his cases and those performed according to his method by other surgeons, have been in a measure successful, so that there is decided encouragement to persevere with the operation in instances in which it appears to be suitable."

IN the CONTEMPORARY REVIEW Mr. Herbert Spencer continues his criticism of Professor Weissmann's theories in a postscript to the essay on "The Inadequacy of Natural Selection," contending that "a right answer to the question whether acquired characters are or are not inherited, underlies right beliefs not only in biology and psychology, but a'so in education, ethics, and politics."

SIR R. S. BALL gives in the same review a picturesque account of the preparations made by astronomers for observing the recent total eclipse of the sun April 15th and 16th, and of the path of the great shadow, oval in form at first, its shortest diameter extending some ninety miles north and south. Professor Pickering was among the first of an ardent corps of astronomers ready to greet it on its arrival on the coast of Chili about half-past seven a.m. Its journey across the wide deserts of the interior, with a speed of something like 3,000 miles an hour is very beautifully described, or rather imagined, to the point of the last glimpse from the Desert of Sahara, where just at the moment of sunset the phase of totality terminated.

